# Reduced Hippocampal Subfield Volume in Schizophrenia and Clinical High-Risk State for Psychosis

**DOI:** 10.3389/fpsyt.2021.642048

**Published:** 2021-03-22

**Authors:** Daiki Sasabayashi, Ryo Yoshimura, Tsutomu Takahashi, Yoichiro Takayanagi, Shimako Nishiyama, Yuko Higuchi, Yuko Mizukami, Atsushi Furuichi, Mikio Kido, Mihoko Nakamura, Kyo Noguchi, Michio Suzuki

**Affiliations:** ^1^Department of Neuropsychiatry, University of Toyama Graduate School of Medicine and Pharmaceutical Sciences, Toyama, Japan; ^2^Research Center for Idling Brain Science, University of Toyama, Toyama, Japan; ^3^Faculty of Medicine, University of Toyama, Toyama, Japan; ^4^Arisawabashi Hospital, Toyama, Japan; ^5^Health Administration Center, University of Toyama, Toyama, Japan; ^6^Department of Radiology, University of Toyama Graduate School of Medicine and Pharmaceutical Sciences, Toyama, Japan

**Keywords:** hippocampal subfield, hippocampal tail, at-risk mental state, schizophrenia, volumetry, magnetic resonance imaging, CA1, molecular layer of the hippocampus

## Abstract

Magnetic resonance imaging (MRI) studies in schizophrenia demonstrated volume reduction in hippocampal subfields divided on the basis of specific cytoarchitecture and function. However, it remains unclear whether this abnormality exists prior to the onset of psychosis and differs across illness stages. MRI (3 T) scans were obtained from 77 patients with schizophrenia, including 24 recent-onset and 40 chronic patients, 51 individuals with an at-risk mental state (ARMS) (of whom 5 subsequently developed psychosis within the follow-up period), and 87 healthy controls. Using FreeSurfer software, hippocampal subfield volumes were measured and compared across the groups. Both schizophrenia and ARMS groups exhibited significantly smaller volumes for the bilateral Cornu Ammonis 1 area, left hippocampal tail, and right molecular layer of the hippocampus than the healthy control group. Within the schizophrenia group, chronic patients exhibited a significantly smaller volume for the left hippocampal tail than recent-onset patients. The left hippocampal tail volume was positively correlated with onset age, and negatively correlated with duration of psychosis and duration of medication in the schizophrenia group. Reduced hippocampal subfield volumes observed in both schizophrenia and ARMS groups may represent a common biotype associated with psychosis vulnerability. Volumetric changes of the left hippocampal tail may also suggest ongoing atrophy after the onset of schizophrenia.

## Introduction

There is increasing evidence supporting that abnormality of the hippocampus, which subserves a range of roles in learning, memory, and emotional regulation (1, 2), functions in the symptomatology and cognitive impairment of schizophrenia (3, 4). Importantly, the hippocampus is not a uniform structure but rather an aggregate of anatomically and functionally different substructures [e.g., the Cornu Ammonis (CA), dentate gyrus (DG), molecular layers, and subiculum; (5)]. Based on the notion of differently affected hippocampal subfields in schizophrenia (6–8), an etiological hypothesis claimed that exaggerated pattern completion induced by aberrant dentate-to-CA3 connections generated psychotic associations (9), whereas another hypothesis argued that hippocampal hypermetabolism originating from CA1 was related to acquired psychotic symptoms and mnemonic interference (10). However, much of the hippocampus-mediated mechanism involved in the onset and progress of psychosis remains unknown. Thus, examining functional or structural abnormalities of the hippocampal subfields, and assessing their potential roles as psychosis biotype constructs may be of interest (11).

A hippocampal volume deficit is among the most robust magnetic resonance imaging (MRI) findings in schizophrenia patients (12–14). However, it remains unclear when such hippocampal abnormalities occur, i.e., either before or after onset, or both, due to inconsistent findings in individuals with an at-risk mental state (ARMS) (15) [reduced hippocampal volume (16–18) or no differences (19–24)] and in patients with schizophrenia [progressive volume loss (19, 25, 26) or no atrophy over time (27–30)]. These discrepancies among previous studies may be partly explained by the possibility that hippocampal reduction exists only in specific subfields (16, 21). However, limited studies of hippocampal subfields reported mixed results [reviewed by Haukvik et al. (31) and Hu et al. (32)], with schizophrenia patients having prominent volume reduction in the CA1 (33) or more widespread reductions in the CA2/3, CA4/DG, presubiculum, subiculum, and CA1 (34, 35). Similar findings were reported in a few studies examining hippocampal subfield volumes in ARMS individuals (23, 36). Hippocampal subfield segmentation on the MRI methodology is under development (37), which may partly explain the heterogeneity of the results. Although diverse relationships between severe psychotic symptoms (34, 38, 39) or poor cognitive performance (34, 36, 40) and volume reductions in CA4/DG, CA2/3, CA1, and subiculum has been reported in schizophrenia patients, it remains unknown whether hippocampal abnormalities are related to subclinical psychotic or cognitive manifestation in ARMS individuals. Further studies are required to examine the hippocampal subfield volume changes in psychotic disorders using a more comprehensive and fine-grained segmentation protocol, ideally in multiple disease phases, including the prodromal stage.

This MRI study investigated volumetric alterations of the hippocampal subfield and their relevance to psychotic symptom or cognitive function in schizophrenia patients, including recent-onset and chronic patients, and ARMS individuals compared with healthy controls. We applied a novel segmentation algorithm using an *ex vivo* atlas (41), which was reported to have superior compatibility with existing histopathological information to the conventional one using only an *in vivo* atlas (42, 43), in order to label the hippocampal subfields. Based on recent MRI findings (23, 33, 36, 40), we predicted that both schizophrenia and ARMS subjects have reduced volumes of the specific hippocampal subfields, but that disease chronicity and/or medication may affect the findings. As hippocampal subfield atrophy and clinical symptoms or socio-cognitive deficits in ARMS were reported to be less severe compared to schizophrenia (36, 44), we also predicted their associations predominantly in schizophrenia.

## Materials and Methods

### Study Participants

Seventy-seven patients with schizophrenia, 51 individuals with ARMS, and 87 healthy control subjects were included in the current study (Table 1). Between December 2013 and August 2019, the study participants were recruited and examined at the clinics of the Department of Neuropsychiatry, Toyama University Hospital.

**Table 1:** Demographic and clinical data of the healthy comparison (HC), at-risk mental state (ARMS), and schizophrenia (Sz) groups.

	**HC**	**ARMS**	**Sz**	**Statistics**
	**(*n* = 87)**	**(*n* = 51)**	**(*n* = 77)**	
Sex, male/female (*n*)	46/41	29/22	39/38	Chi-square = 0.48, *p* = 0.788
Age (years)	26.3 ± 3.9	18.3 ± 4.2	28.8 ± 9.4	*F*_(2, 214)_ = 41.59, *p* < 0.001; ARMS < HC < Sz
Height (cm)	165.7 ± 8.3	164.1 ± 8.0	164.3 ± 8.8	*F*_(2, 214)_ = 0.77, *p* = 0.465
Intracranial volume (ml)	1553 ± 126	1485 ± 144	1501 ± 168	*F*_(2, 214)_ = 3.74, *p* = 0.025^a^; ARMS < HC, Sz
JART-IQ^b^	110.0 ± 6.8	97.3 ± 9.3	101.2 ± 8.7	*F*_(2, 181)_ = 43.06, *p* < 0.001; ARMS < Sz < HC
Handedness (right/left/mixed)	60/8/19	31/3/17	63/2/12	Chi-square = 9.33, *p* = 0.053
Socioeconomic status	6.3 ± 0.8	3.1 ± 1.4	4.4 ± 1.3	*F*_(2, 214)_ = 131.15, *p* < 0.001; ARMS < Sz < HC
Parental socioeconomic status^c^	5.9 ± 0.9	4.9 ± 0.9	4.9 ± 1.3	*F*_(2, 213)_ = 21.36, *p* < 0.001; ARMS, Sz < HC
Age at onset (years)			22.8 ± 8.1	
Duration of psychosis (years)			5.6 ± 6.5	
Medication dose (HPD equivalent, mg/day)		3.0 ± 3.2 (*n* = 11)	10.6 ± 8.3 (*n* = 65)	*F*_(1, 75)_ = 8.73, *p* = 0.004; ARMS < Sz
Medication type (atypical/typical/mixed)		10/1/0	56/0/9	Chi-square = 139.39, *p* < 0.001
Duration of medication (years)		0.6 ± 0.8 (*n* = 6)	6.1 ± 6.8 (*n* = 56)	*F*_(1, 61)_ = 3.79, *p* = 0.056
PANSS positive		12.3 ± 3.4	15.5 ±6.3	*F*_(1, 124)_ = 10.47, *p* = 0.002; ARMS < Sz
PANSS negative		16.4 ± 6.9	18.2 ± 7.5	*F*_(1, 124)_ = 1.98, *p* = 0.162
PANSS general		31.9 ± 7.9	35.0 ± 11.5	*F*_(1, 124)_ = 2.71, *p* = 0.102
**CAARMS subscale scores**				
Unusual thought global rating scale		3.6 ± 1.4		
Unusual thought frequency scale		3.6 ± 1.9		
Non-Bizarre ideas global rating scale		3.9 ± 1.1		
Non-Bizarre ideas frequency scale		4.4 ± 1.3		
Perceptual abnormalities global rating scale		3.1 ± 1.6		
Perceptual abnormalities frequency scale		3.1 ± 1.9		
Disorganized speech global rating scale		2.5 ± 1.3		
Disorganized speech frequency scale		4.1 ± 2.4		
**BACS subdomain z-scores**				
Verbal memory		−0.7 ± 1.4	−1.3 ± 1.4	*F*_(1, 112)_ = 6.05, *p* = 0.015; Sz < ARMS
Working memory		−0.8 ± 1.3	−0.9 ± 1.3	*F*_(1, 112)_ = 0.16, *p* = 0.692
Motor function		−0.9 ± 1.3	−2.0 ± 1.5	*F*_(1, 112)_ = 19.59, *p* < 0.001; Sz < ARMS
Verbal fluency		−0.9 ± 1.4	−0.9 ± 1.1	*F*_(1, 112)_ = 0.024, *p* = 0.877
Attention and processing speed		−0.3 ± 1.4	−1.2 ± 1.3	*F*_(1, 112)_ = 12.03, *p* < 0.001; Sz < ARMS
Executive function		−0.4 ± 1.3	−0.7 ± 1.8	*F*_(1, 112)_ = 0.87, *p* = 0.354
BACS mean z-score		−0.7 ± 1.0	−1.2 ± 1.0	*F*_(1, 112)_ = 7.13, *p* = 0.009; Sz < ARMS
SCoRS global rating score		5.3 ± 2.2	5.0 ± 2.5	*F*_(1, 102)_ = 0.43, *p* = 0.516
SOFAS		50.2 ± 10.5	47.3 ± 14.3	*F*_(1, 87)_ = 1.19, *p* = 0.279

Values represent the mean ± SD unless otherwise stated.

ARMS, At-Risk Mental State; BACS, Brief Assessment of Cognition in Schizophrenia; CAARMS, Comprehensive Assessment of At-Risk Mental State; IQ, Intelligence Quotient; JART, Japanese version of National Adult Reading Test; HC, healthy controls; HPD, haloperidol; PANSS, Positive and Negative Syndrome Scale; SCoRS, Schizophrenia Cognition Rating Scale; SOFAS, Social and Occupational Functioning Assessment Scale; Sz, schizophrenia.

a Age was used as a covariate.

b Data missing for 33 subjects.

c *Data missing for one subject*.

The schizophrenia patients were assessed by the Structured Clinical Interview for DSM-IV Axis I Disorders Patient Edition (SCID-I/P) (45) and a detailed chart review, and fulfilled both the DSM-IV-TR (46) and DSM-5 (47) criteria. Recent-onset schizophrenia (ROSz) patients were defined by a duration of psychosis <1 year (*n* = 24, age = 24.5 ± 10.1 years, duration of psychosis = 0.4 ± 0.2 years) (48, 49), whereas chronic schizophrenia patients were defined as those with a duration of psychosis >3 years (*n* = 40, age = 32.4 ± 8.5 years, duration of psychosis = 9.9 ± 6.4 years) (50). As an additional analysis, we also defined the chronic schizophrenia patients as those with a duration of psychosis > 10 years (*n* = 16, age = 36.1 ± 7.2 years, duration of psychosis = 16.2 ± 5.6 years) to limit them to more chronic patients. Sixty-five patients with schizophrenia were receiving antipsychotics at the time of MRI. They were treated with risperidone (*n* = 7), paliperidone (*n* = 4), olanzapine (*n* = 25), quetiapine (*n* = 4), aripiprazole (*n* = 17), perospirone (*n* = 6), blonanserin (*n* = 9), zotepine (*n* = 1), clozapine (*n* = 1), haloperidol (*n* = 2), levomepromazine (*n* = 6), and/or fluphenazine (*n* = 1).

Through a local early intervention service in Toyama (51), ARMS individuals who were diagnosed by the Japanese version of the Comprehensive Assessment of At Risk Mental States (CAARMS) (15, 52) were recruited. All 51 ARMS individuals didn't exceed the threshold for psychosis on the CAARMS at baseline (Table 1). The ARMS individuals were prospectively followed (mean = 3.7 years, *SD* = 3.0 years), and subdivided into five individuals (9.6%) who later developed psychosis (ARMS-P) and 28 who did not develop psychosis during clinical follow-up of at least 2 years (ARMS-NP). Based on the DSM-IV-TR criteria, all psychotic disorders in ARMS-P subjects were diagnosed as schizophrenia. Regarding psychiatric comorbidities, ARMS subjects were also diagnosed with pervasive developmental disorders (PDD) (*n* = 5), attention-deficit and disruptive behavior disorders (*n* = 1), depressive disorders (*n* = 6), anxiety disorders (*n* = 8), dissociative disorders (*n* = 1), eating disorders (*n* = 1), adjustment disorders (*n* = 9), schizotypal personality disorders (*n* = 3), or avoidant personality disorders (*n* = 1). At the timing of MRI, 11 subjects (21.6%) were receiving a low dosage of antipsychotics for their severe psychiatric conditions in accordance with the clinical guidelines for early psychosis (53). Simultaneously, 5 subjects (9.8%) were taking antidepressants (imipramine equivalent doses = 112.5 ± 65.0 mg/day), and 14 subjects (27.5%) were taking anxiolytics (diazepam equivalent doses = 5.1 ± 2.2 mg/day). Omega-3 fatty acids were not used in any subjects.

Healthy control subjects with no personal or family (first-degree relatives) history of psychiatric diseases who were screened by the SCID-I Non-patient Edition (45) were recruited from hospital staff, University students, and members of the local community.

All participants in the present study were physically healthy at the time of MRI and had no lifetime history of serious head trauma, neurological illness, substance abuse, steroid use, or other serious physical diseases. One hundred and sixty-one of the 216 subjects were also included in our previous study that investigated subregional volumes of the thalamus and basal ganglia in schizophrenia and ARMS (54). The Committee on Medical Ethics of Toyama University approved this study. Written informed consent was received from all study participants. If the participants were under the age of 20, their parent or guardian also provided written consent.

### Clinical Assessment

Clinical symptoms of the schizophrenia and ARMS subjects were rated by the Positive and Negative Syndrome Scale (PANSS) (55), whose scores consisted of the positive items, negative items, and general psychopathology. Cognitive assessments were conducted using the Brief Assessment of Cognition in Schizophrenia (BACS) (56, 57). The BACS scores from their six subdomains (verbal memory, working memory, motor speed, verbal fluency, attention, and executive function) were standardized by calculating z-scores, where the mean score of the healthy Japanese was set to zero and the standard deviation was set to one (58). The Schizophrenia Cognition Rating Scale (SCoRS) (59–61) were also conducted to measure the cognitive abilities related to daily-living functioning or functional capacity. Among 20 items of the SCoRS, global rating scale (range 1–10, higher ratings mean greater impairment in daily living skills) was adapted as a representative value. Social functioning was evaluated by the Social and Occupational Functioning Assessment Scale (SOFAS) (62), whose score (range 0–100, higher ratings mean better functioning) corresponded to the social functioning domain of the Global Assessment of Functioning Scale in the DSM-IV-TR (46). All assessments were administered by experienced psychiatrists and trained psychologists.

### MRI

Study participants were scanned using a 3-T Magnetom Verio (Siemens Medical System, Inc., Erlangen, Germany) with a three-dimensional magnetization-prepared rapid gradient echo (MPRAGE) sequence yielding 176 contiguous T1-weighted slices of 1.2-mm thickness in the sagittal plane. The imaging parameters were as follows: repetition time = 2,300 ms, echo time = 2.9 ms, flip angle = 9°, field of view = 256 mm, and matrix size = 256 × 256. The voxel size was 1.0 × 1.0 × 1.2 mm.

### Measurement of Hippocampal Subfields

Preprocessing of the T1-weighted images, including the correction for intensity non-uniformity in MRI data (63), was performed using the FreeSurfer pipeline (version 6.0, http://surfer.nmr.mgh.harvard.edu) (64, 65). One trained researcher (RY) blinded to the subjects' identities visually inspected all reconstructed images, and manually edited them to improve their subcortical and temporolimbic segmentations. The hippocampal region was automatically segmented into 12 different subfields using a new algorithm, which was based on a computational atlas assembled from *ex vivo* MRI data of post-mortem medial temporal tissue and *in vivo* MRI data informing about neighboring extrahippocampal structures (41). All subfield outputs were also visually inspected to ensure no robust mislabeling. We measured the intracranial volume (ICV), and volume of the entire hippocampus and 12 hippocampal subfields: hippocampal tail, subiculum, CA1, hippocampal fissure, presubiculum, parasubiculum, molecular layer hippocampus (HP), granule cell and molecular layer of the dentate gyrus (GC-ML-DG), CA3, CA4, fimbria, and hippocampus-amygdala-transition-area (HATA).

### Statistical Analysis

Clinical and demographic differences among groups were examined by one-way analysis of variance (ANOVA) or chi-square test.

Absolute regional volumes were analyzed using the repeated measures multivariate analysis of variance (MANCOVA), with age and ICV as covariates, diagnosis (e.g., healthy controls vs. ARMS vs. schizophrenia, ARMS-P vs. ARMS-NP, ROSz vs. chronic schizophrenia, and ARMS vs. ROSz vs. chronic schizophrenia) and sex as between-subject factors, and hemisphere and hippocampal subfields (12 regions) as within-subject variables. We assessed the effects of subfield by lower order MANCOVA only when we detected significant diagnosis-by-subfield-by-hemisphere interactions (Table 2) in order to prevent possible type I errors. *Post-hoc* Newman-Keuls tests were employed to follow-up the significant main effects or interactions.

**Table 2:** Absolute volume of the hippocampal subfields in the HC, ARMS, and Sz groups.

**Region of Interest** **(mm^**3**^)**	**HC (*n* = 87)**	**ARMS (*n* = 51)**	**Sz (*n* = 77)**	**Multivariate analysis of covariates**		***Post-hoc*** **tests**	
	**(Male 46,** **Female 41)**	**(Male 29,** **Female 22)**	**(Male 39,** **Female 38)**	**Diagnosis × Subfield × Hemisphere**		**Sz vs. HC**	**ARMS vs. HC**
	**Mean ± SD**	**Mean ± SD**	**Mean ± SD**	***F*_**(22, 2299)**_**	***P***	***P***	***P***
Entire hippocampus				1.79	**0.01**		
Left	3523.5 ± 310.5	3403.5 ± 340.3	3378.0 ± 312.2				
Right	3615.6 ± 343.7	3429.0 ± 312.8	3495.5 ± 361.8				
Hippocampal tail				–	–		
Left	551.8 ± 65.9	522.7 ± 53.6	520.0 ± 58.3			**1.76 × 10**^**−5**^	**5.67 × 10**^**−5**^
Right	570.6 ± 70.5	542.7 ± 67.6	554.6 ± 55.6			0.08	**5.51 × 10**^**−4**^
Subiculum				–	–		
Left	445.5 ± 47.9	430.3 ± 50.8	432.3 ± 47.0			0.12	0.10
Right	454.8 ± 48.3	424.4 ± 48.4	439.9 ± 50.1			0.06	**8.30 × 10**^**−5**^
CA1				–	–		
Left	635.9 ± 72.4	619.8 ± 69.5	612.3 ± 63.5			**1.15 × 10**^**−3**^	**1.54 × 10**^**−2**^
Right	676.9 ± 92.2	643.5 ± 73.5	652.5 ± 75.5			**2.40 × 10**^**−4**^	**2.30 × 10**^**−5**^
Hippocampal fissure							
Left	150.5 ± 25.4	154.0 ± 29.5	153.1 ± 26.6			0.70	0.86
Right	144.8 ± 21.8	143.5 ± 23.1	154.2 ± 26.8			0.62	0.85
Presubiculum				–	–		
Left	320.1 ± 33.6	311.4 ± 41.0	306.3 ± 39.0			0.23	0.38
Right	313.1 ± 35.5	296.4 ± 34.6	299.8 ± 42.2			0.34	0.19
Parasubiculum				–	–		
Left	65.2 ± 10.1	63.6 ± 9.2	60.4 ± 9.9			0.98	0.82
Right	61.3 ± 9.8	59.8 ± 8.2	57.3 ± 9.6			1.00	1.00
Molecular layer HP				–	–		
Left	576.1 ± 56.0	558.4 ± 59.7	552.7 ± 54.8			**7.77 × 10**^**−3**^	0.06
Right	597.6 ± 65.6	563.5 ± 54.8	575.5 ± 60.9			**2.56 × 10**^**−3**^	**1.97 × 10**^**−5**^
GC-ML-DG				–	–		
Left	303.5 ± 33.9	295.3 ± 37.4	291.6 ± 35.6			0.55	0.73
Right	307.8 ± 36.0	292.1 ± 30.4	298.2 ± 40.6			0.60	0.27
CA3				–	–		
Left	200.4 ± 25.8	195.4 ± 27.1	195.4 ± 26.3			0.88	0.74
Right	207.4 ± 29.6	198.8 ± 26.8	206.2 ± 33.4			0.85	0.57
CA4				–	–		
Left	260.1 ± 29.0	253.2 ± 32.7	249.0 ± 29.1			0.34	0.55
Right	260.4 ± 30.0	248.4 ± 26.3	253.8 ± 34.9			0.58	0.46
Fimbria				–	–		
Left	102.8 ± 16.8	94.1 ± 16.8	99.5 ± 20.9			0.62	0.69
Right	103.5 ± 18.1	96.3 ± 17.2	97.9 ± 22.5			0.84	0.82
HATA				–	–		
Left	62.2 ± 7.5	59.2 ± 6.0	58.5 ± 8.4			1.00	1.00
Right	62.3 ± 7.4	59.4 ± 8.0	59.9 ± 9.2			1.00	1.00

ARMS, at-risk mental state; CA, Cornu Ammonis; GC-ML-DG, granule cell and molecular layer of the dentate gyrus; HATA, hippocampus-amygdala-transition-area; HC, healthy controls; HP, hippocampus; Sz, schizophrenia.

*Bold font indicates statistical significance*.

Test-retest reliability of FreeSurfer automated hippocampal subfield segmentation has been established using 3T-MRI data (66, 67). For validation analyses, however, we also combined parts of the subfields to set up the merged hippocampal subfields, such as CA1, subiculum_combined_ (subiculum + presubiculum + parasubiculum), and other (GC-ML-DG + CA3 + CA4) subfields on the basis of previous studies (68, 69). Using the same repeated measures MANCOVA model, absolute regional volumes of these merged subfields were analyzed among the schizophrenia, ARMS, and control groups.

Relationships between the absolute volume of the hippocampal subfields with significant group differences (i.e., hippocampal tail, subiculum, CA1, and molecular layer HP; Table 2) and clinical or socio-cognitive variables [e.g., age at onset, duration of psychosis, medication dose, duration of medication, PANSS (positive, negative, and general), BACS (mean z-scores), SCoRS global rating score, and SOFAS] in the schizophrenia and ARMS groups were explored by Pearson's partial correlation coefficients controlled for age, sex, and ICV.

The significance threshold was set at *p* < 0.05 (two-sided). For correlation analyses, a Bonferroni correction was applied to correct for multiple comparisons.

## Results

### Sample Characteristics

Demographic and clinical characteristics of the sample are summarized in Table 1. Groups were matched for sex, height, and handedness, but there were significant differences in age, ICV, premorbid Intelligence Quotient, and personal/parental socioeconomic status. The schizophrenia patients were characterized by higher PANSS positive scores, lower BACS measures, and greater amounts of antipsychotics than ARMS individuals.

### Volumetric Analyses

On comparison among the schizophrenia, ARMS, and control groups, MANCOVA of the hippocampal volume revealed a significant diagnosis-by-subfield-by-hemisphere interaction. We therefore separately evaluated the group differences in hippocampal subfields for each hemisphere. Compared with controls, the schizophrenia group had a smaller volume in the bilateral CA1, bilateral molecular layer HP, and left hippocampal tail, and the ARMS group had a smaller volume in the bilateral hippocampal tail, bilateral CA1, right subiculum, and right molecular layer HP (Table 2). However, the hippocampal volumes did not differ between the schizophrenia and ARMS groups. ARMS subsample without comorbid PDD diagnosis (*n* = 46) also exhibited a smaller volume in the hippocampal tail (*p* = 3.20 × 10^−5^ for left side and *p* = 6.78 × 10^−5^ for right side), CA1 (*p* = 2.29 × 10^−2^ for left side and *p* = 9.54 × 10^−6^ for right side), and subiculum (*p* = 3.02 × 10^−2^ for left side and *p* = 6.69 × 10^−5^ for right side) bilaterally, as well as in the right molecular layer HP (*p* = 1.15 × 10^−5^) compared with controls (Supplementary Table 1).

There were no significant differences in the hippocampal volumes between the ARMS-P and -NP groups (Supplementary Table 2).

On comparison between the ROSz and chronic schizophrenia groups, a significant diagnosis-by-subfield-by-hemisphere interaction was observed by MANCOVA [*F*_(11, 660)_ = 3.58, *p* < 0.001]. *Post-hoc* analyses demonstrated that the left hippocampal tail was significantly reduced in chronic schizophrenia patients compared with ROSz patients (*p* = 1.58 × 10^−4^) (Supplementary Table 3). Similarly, re-defined chronic schizophrenia patients (duration of psychosis >10 years) exhibited a significant volume reduction only in the left hippocampal tail compared with ROSz patients (*p* = 2.42 × 10^−4^) (Supplementary Table 4).

Direct comparison among the ARMS, ROSz, and chronic schizophrenia groups showed a significant diagnosis-by-subfield-by-hemisphere interaction [*F*_(22, 1199)_ = 3.19, *p* < 0.001], and the *post-hoc* tests indicated that the left hippocampal tail was significantly reduced in chronic schizophrenia group compared with ROSz (*p* = 2.82 × 10^−5^) and ARMS (*p* = 8.28 × 10^−3^) groups, as well as in ARMS group compared with ROSz (*p* = 3.81 × 10^−2^) group (Supplementary Table 5).

For the analysis of merged hippocampal subfields, a significant diagnosis-by-hemisphere interaction was observed by MANCOVA [*F*_(2, 209)_ = 5.12, *p* = 0.01]. *Post-hoc* analyses demonstrated that sum of the merged hippocampal subfield of the right hemisphere was significantly reduced in ARMS individuals compared with controls (*p* = 5.80 × 10^−3^) (Supplementary Table 6). However, MANCOVA showed no significant interactions involving diagnosis-by-subfield, supporting the utility of more detailed subfield analyses.

The results of these comparisons remained essentially the same even when medication (dosage and duration) was included as a covariate.

### Correlation Analyses

**Figure 1:**
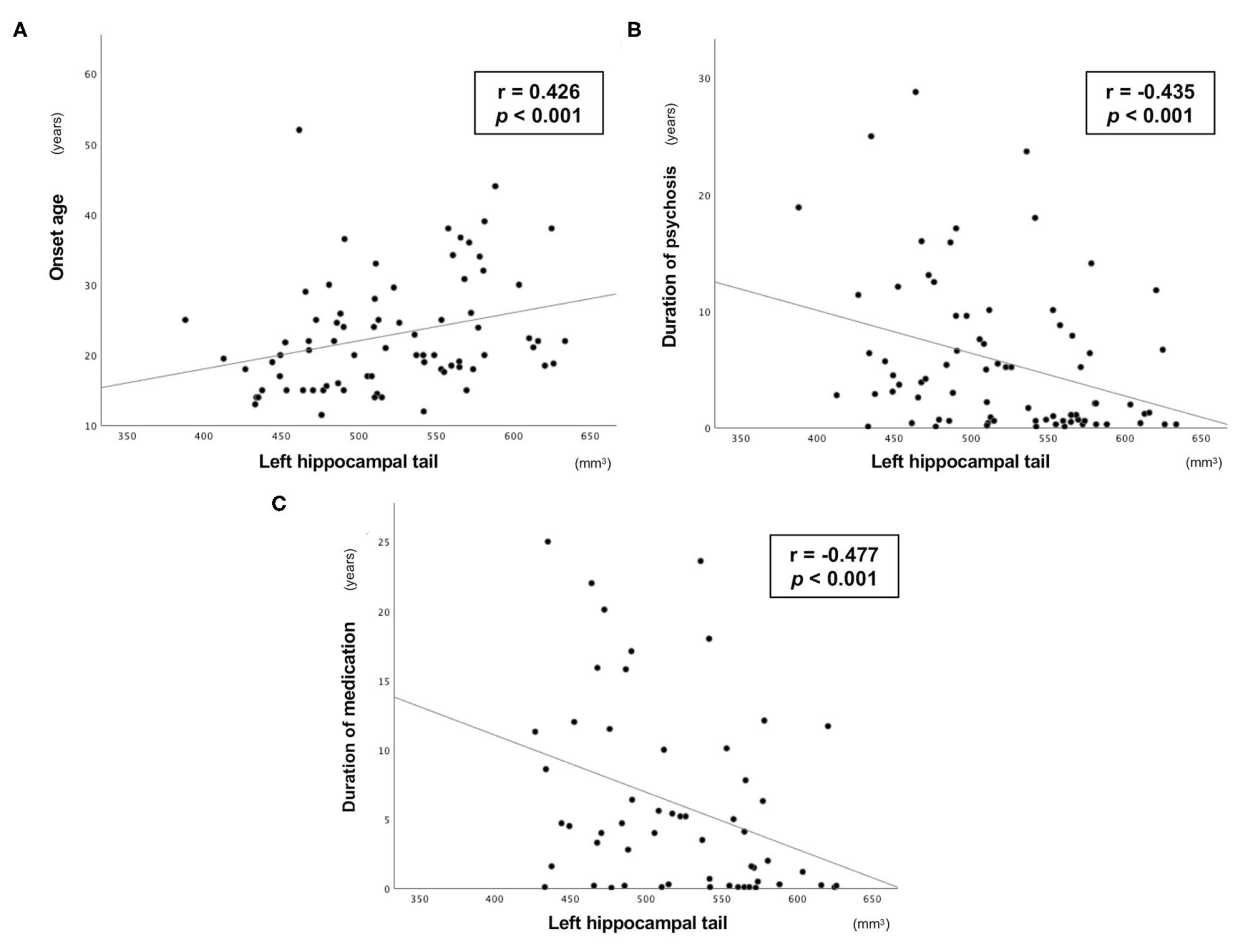
Relationship between the absolute volume of left hippocampal tail and clinical variables in the Sz group. Relationship between the absolute volume of the left hippocampal tail and **(A)** onset age, **(B)** duration of psychosis, and **(C)** duration of medication in patients with schizophrenia.

The left hippocampal tail volume was positively correlated with onset age and negatively correlated with duration of psychosis in patients with schizophrenia (Figure 1, Table 3). In the schizophrenia group, volume reduction of the left hippocampal tail was significantly associated with long-term medication use, whereas the hippocampal subfield volume was not associated with antipsychotic medication dosage (Figure 1, Table 3). In ARMS individuals, we found no significant relationship between the hippocampal volume and clinical or socio-cognitive variables.

**Table 3:** Relationships between the hippocampal volume and clinical variables in the schizophrenia and ARMS groups.

	**ARMS**												**Sz**									
	**Left** **hippocampal** **tail**		**Right** **hippocampal** **tail**		**Right** **subiculum**		**Left CA1**		**Right CA1**		**Right** **molecular** **layer HP**		**Left** **hippocampal** **tail**		**Left CA1**		**Right CA1**		**Left** **molecular** **layer HP**		**Right** **molecular** **layer HP**	
	***rho***	***p***	***rho***	***p***	***rho***	***p***	***rho***	***p***	***rho***	***p***	***rho***	***p***	***rho***	***p***	***rho***	***p***	***rho***	***p***	***rho***	***p***	***rho***	***p***
Age at onset (years)	–	–	–	–	–	–	–	–	–	–	–	–	0.426	**1.52 × 10**^**−4**^	−0.020	0.866	−0.081	0.491	0.040	0.734	0.019	0.872
Duration of psychosis (years)	–	–	–	–	–	–	–	–	–	–	–	–	−0.435	**1.21 × 10**^**−4**^	0.009	0.940	0.035	0.772	−0.067	0.573	−0.065	0.587
Meducation dose (HPD equiv., mg/day)	0.606	0.111	0.194	0.645	0.084	0.843	−0.056	0.896	−0.381	0.351	−0.189	0.654	0.019	0.882	−0.084	0.516	0.095	0.464	−0.177	0.169	0.035	0.787
Duration of medication (years)	0.007	0.996	0.837	0.369	−0.687	0.518	−0.981	0.126	−0.824	0.384	−0.983	0.119	−0.477	**3.00 × 10**^**−4**^	−0.028	0.843	−0.054	0.702	−0.093	0.509	−0.122	0.384
PANSS positive	−0.002	0.987	0.051	0.734	0.101	0.499	−0.018	0.906	0.175	0.240	0.034	0.821	−0.243	0.040	−0.104	0.383	−0.036	0.766	−0.062	0.605	−0.129	0.281
PANSS negative	0.249	0.091	0.193	0.195	0.268	0.069	0.320	0.028	0.286	0.052	0.288	0.049	−0.007	0.956	0.015	0.897	0.123	0.304	0.084	0.482	0.100	0.405
PANSS general	0.010	0.949	0.033	0.827	0.089	0.550	0.167	0.261	0.228	0.123	0.123	0.411	−0.098	0.414	−0.162	0.174	0.058	0.626	−0.059	0.622	−0.023	0.848
BACS mean z-score	−0.056	0.716	−0.176	0.249	−0.001	0.994	−0.271	0.072	0.048	0.755	−0.017	0.913	−0.107	0.410	0.029	0.825	0.006	0.961	−0.099	0.445	−0.004	0.975
SCoRS global rating score	−0.010	0.944	−0.082	0.578	0.145	0.324	0.082	0.581	−0.063	0.668	−0.077	0.604	0.161	0.270	−0.183	0.209	−0.184	0.207	−0.144	0.325	−0.197	0.176
SOFAS	0.002	0.990	−0.086	0.601	0.035	0.831	−0.317	0.049	−0.115	0.485	−0.047	0.777	−0.135	0.387	0.136	0.385	−0.059	0.708	0.084	0.591	−0.061	0.700

ARMS, at-risk mental state; BACS, Brief Assessment of Cognition in Schizophrenia; CA, Cornu Ammonis; HP, hippocampus; HPD, haloperidol; PANSS, Positive and Negative Syndrome Scale; SCoRS, Schizophrenia Cognition Rating Scale; SOFAS, Social and Occupational Functioning Assessment Scale; Sz, schizophrenia.

*Bold font indicates statistical significance after Bonferroni's correction for multiple comparisons [seven subfields by 10 clinical variables; p < 0.000714 (0.05/70)]*.

## Discussion

In the present MRI study, we have investigated hippocampal subfield volumes based on a reliable *ex vivo* atlas cross-sectionally across multiple stages of psychosis. The schizophrenia and ARMS groups had significantly smaller volumes of the CA1, hippocampal tail, and molecular layer HP than healthy controls, suggesting that hippocampal abnormalities in these specific subfields represent a static vulnerability marker of psychosis. On the other hand, the volume loss in the left hippocampal tail preferentially observed in the chronic stage of psychosis, which was related to early onset age and long-term duration of psychosis, may reflect a regional progressive pathological process after onset.

Our finding of reduced hippocampal volume, especially in the CA1, hippocampal tail, and molecular layer HP, was partly consistent with four previous studies (23, 33, 43, 70) in psychotic disorders that assessed hippocampal subfields using a recent version of segmentation by Iglesias et al. (41). On the other hand, previous studies (34, 35, 39) mainly employing an earlier version of segmentation by Leemput et al. (42) reported widespread volume reductions centered on the CA2/3, CA4/DG, and subiculum. Different segmentation methods among the studies may be partly responsible for these discrepancies; the segmentation protocol by Leemput et al. (42) may have underestimated CA1 volumes and overestimated CA2/3 or subiculum volumes compared with manual demarcation (71, 72). In addition, although the relationship between hippocampal subfield morphology and antipsychotic medication has not been well-documented (31), we cannot exclude the potential confounding effects of antipsychotic medication on the results, in consideration of experimental findings of alterations in hippocampal neurogenesis (73) and hippocampal volumes (74) after antipsychotic treatment. Indeed, we noted a relationship between the hippocampal tail and medication duration, but not medication dosage. The discrepancy might be partly due to the inseparable effects of duration of medication and psychosis, or to the opposite effects of medication dosage on hippocampal anatomy in acute and long-term treatment (75). As the group difference remained significant even when we added medication duration and dosage as covariates in the analytical model, reduced volume of the hippocampal subfields in our schizophrenia cohort cannot be explained only by antipsychotic drug action. Although we failed to detect a significant relationship between hippocampus atrophy and clinical symptoms or cognitive deficits, further studies are required to clarify each specialized role of functional/structural abnormalities of the CA1, molecular layer HP, and hippocampal tail in the pathophysiology of schizophrenia.

Partially consistent with a previous study of an ARMS cohort (23, 36), clinically high-risk subjects for psychosis demonstrated reduced volumes in the CA1, molecular layer HP, hippocampal tail, and subiculum, most of which were also observed in schizophrenia patients. Because the exclusion of ARMS individuals with PDD diagnosis did not change the conclusion of the study, the hippocampal findings in ARMS may not be explained only by the coexistence of PDD. However, volumes of these subfields did not differ between schizophrenia and ARMS subjects or between ARMS individuals with and without subsequent transition to psychosis in contrast to a few previous findings (36, 76). As rather small sample size of the ARMS-P individuals (*n* = 5) in our cohort could partly explain such discrepancy, their role as a biological discrimination for subsequent psychosis should be further tested in a larger ARMS-P cohort. Reduced hippocampal subfield volumes commonly observed in schizophrenia and ARMS groups should represent a common biotype involved in vulnerability to psychosis. Recently, approaches that can alter some biotypes, such as deficits in hippocampal perfusion or sensory gating (76–78), have been considered as early interventions for psychosis (79, 80). As aerobic exercise and cognitive enhancement therapy can prevent the hippocampal volume decreases over time in early psychosis (81, 82), this biotype may be one of the target candidates for prophylactic treatment in the future.

In contrast to the conventional notion that hippocampal abnormality is a stable feature of schizophrenia (27–30), the combination of the more marked hippocampal tail atrophy in chronic patients relative to recent-onset patients and its relationship with onset age or duration of psychosis suggests a progressive decrease in the hippocampal subfield volume. Direct group comparison also showed the role of illness stages on the hippocampal tail (ROSz > ARMS > chronic schizophrenia), but this result should be interpreted with cautions due to relatively small sample size of ROSz group and significant group difference in age (although statistically controlled). Although the hippocampal tail has not been well-investigated neuroanatomically (41), previous cross-sectional MRI studies reported that reduced volume of this subfield was observed in schizophrenia patients with a longer duration of psychosis (33, 40) in contrast with those with a shorter duration of psychosis (33, 70). Conversely, two longitudinal studies (33, 39) demonstrated that patients with schizophrenia exhibited progressive volume loss in several hippocampal subfields, such as CA1-4, DG, and subiculum, as opposed to putative ongoing atrophy only in the hippocampal tail in this cohort. These studies (33, 39) supported the progressive pathology of schizophrenia (19, 25, 26) by confirming that the symptomatic deterioration was synchronized with the decrease in hippocampal volume, although they had limitations; their cohorts were characterized by a relatively small sample size, short-term follow-up period, and mixture of recent-onset and chronic patients. Future large-scale longitudinal studies are required to directly examine the trajectory of the hippocampal subfield atrophy at varying stages of psychotic disorders, focusing on the spatial distribution of subregional deficits.

Although the current MRI study was unable to sufficiently clarify the etiological role of the hippocampus in psychotic disorder, our finding of focal shrinkage in the CA1 and subiculum [molecular layer HP was classified as part of the subiculum or CA fields in most previous segmentations (41, 42)] that developed around onset partly supports the hippocampal hyperactivity models (83, 84). Among them, Small et al. (10) proposed the early involvement of CA1 (and subiculum) in the pathophysiological process responsible for psychosis because it has greater expression of the N-methyl-D-aspartate (NMDA) receptor (85) and may be especially vulnerable to glutamate-mediated neurotoxicity (86). Therefore, excess extracellular glutamate that accumulates preferentially in the CA1/subiculum in the early disease stage affects metabolic demand and blood flow, and causes eventual volume loss in the corresponding region (7, 76, 87). Dysfunction of gamma-aminobutyric acid (GABA)-ergic interneurons, which were proposed to underlie the metabolic and structural alterations in these hippocampal subfields, may propagate to other hippocampal subfields and drive feedforward excitation of the hippocampal trisynaptic circuit (88, 89), leading to the clinical features of schizophrenia and cognitive impairments (90–93). Furthermore, the finding of reduced NMDA receptor related proteins only in the dentate molecular layer in schizophrenia post-mortem brains may imply the specific role of molecular layer in this cascade (94). Alternatively, we previously suggested that only the hippocampal tail exhibits progressive atrophy across the disease stages in contrast to the assumption that hippocampal subfield volume losses extend along the trisynaptic pathway [e.g., CA3-4 and DG; (33)]. In this regard, even though demarcation of the hippocampal tail was slightly different from that in the present study, the cumulative adverse effects of psychotic episodes on the left hippocampal tail have been reported (95). In methylazoxymethanol acetate treated rats as a developmental disruption model of schizophrenia (96), a reduction in synaptic innervation and excitatory synaptic transmission was observed especially in the dorsal hippocampus (97). Thus, the nature of static or progressive structural/functional changes of the hippocampus, particularly in the posterior portion where fewer studies have focused, remains unclear.

Some limitations to the present study should be delineated. First, in order to label the hippocampal subfields, we adopted a new and validated segmentation protocol (41), but it was based on only a T1 sequence, as employed in most previous studies (23, 43, 70). We may be able to obtain more reliable segmentation utilizing an additional T2 sequence (98). Second, hippocampal morphometric changes may be affected not only by intrinsic factors of psychosis, but also by potential confounding factors such as antipsychotics (99), comorbid anxiety and depression (100), and prolonged stress (101). Future studies should try to replicate the current hippocampal findings in antipsychotic-naïve schizophrenia patients whose comorbid symptoms are well-managed. Thirdly, there are no consensus operational definitions for “resent-onset” or “chronic” schizophrenia [e.g., DSM-IV-TR; (46)]. Although our results did not change significantly between different chronic definitions, potential role of illness stages on the hippocampal volume should be further tested in future longitudinal studies in various illness stages. Fourthly, although the established reliability of automated subfield segmentation (66, 67), our results of significant group difference predominantly in relatively large hippocampal subfields (CA1, molecular layer HP, and hippocampal tail) may raise the possibility of technical issue that prevents accurate group comparison of smaller subfields. Lastly, volume reduction of the hippocampal subfields, especially in the CA1, was also noted in other neuropsychiatric illnesses such as post-traumatic stress disorder, major depressive disorder, and bipolar disorder (102, 103). On the other hand, volume reductions in the CA2/3 and presubiculum were more pronounced in schizophrenia than in bipolar disorder (31), possibly contributing to discrimination among psychiatric disorders. Thus, whether our hippocampal findings belong to a common biotype across psychiatric disorders or a distinct biotype of the schizophrenia spectrum should be investigated.

In conclusion, this MRI study demonstrated that both schizophrenia and ARMS groups exhibit smaller hippocampal volumes, especially in CA1, hippocampal tail, and molecular layer HP subfields. Reduced volume of the left hippocampal tail in schizophrenia was associated with illness chronicity and antipsychotic medication. The hippocampal subfield atrophy may represent a potential biotype that accounts for psychosis vulnerability, but further studies are needed to clarify how it is involved in the formation and development of psychotic disorders.

## Data Availability Statement

The datasets utilized for this article are not available immediately because we do not have permission to share them. Requests to access the datasets should be directed to Daiki Sasabayashi, ds179@med.u-toyama.ac.jp.

## Ethics Statement

The studies involving human participants were reviewed and approved by the Committee on Medical Ethics of Toyama University. Written informed consent to participate in this study was provided by the participants' legal guardian/next of kin.

## Author Contributions

DS, TT, YT, and MS conceived the present study and its methods. DS conducted statistical analyses and wrote the manuscript. DS, SN, YH, YM, AF, MK, and MN recruited participants, and were involved in clinical and diagnostic assessments. DS and RY analyzed MRI data. KN provided technical support for MRI and data processing. DS, AF, MN, and TT managed MRI and clinical data. TT, YT, and MS contributed to the writing and editing of the manuscript. All authors contributed to and approval the final manuscript.

## Conflict of Interest

The authors declare that the research was conducted in the absence of any commercial or financial relationships that could be construed as a potential conflict of interest.

## References

[1] Kim JJ Diamond DM. The stressed hippocampus, synaptic plasticity and lost memories. Nat Rev Neurosci. (2002) 3:453–62. 10.1038/nrn849 12042880

[2] Sweeney P Yang Y. Neural circuit mechanisms underlying emotional regulation of homeostatic feeding. Trends Endocrinol Metab. (2017) 28:437–48. 10.1016/j.tem.2017.02.006 28279562 PMC5438765

[3] Harrison PJ. The hippocampus in schizophrenia: a review of the neuropathological evidence and its pathophysiological implications. Psychopharmacology. (2004) 174:151–62. 10.1007/s00213-003-1761-y 15205886

[4] Lieberman JA Girgis RR Brucato G Moore H Provenzano F Kegeles L. Hippocampal dysfunction in the pathophysiology of schizophrenia: a selective review and hypothesis for early detection and intervention. Mol Psychiatry. (2018) 23:1764–72. 10.1038/mp.2017.249 29311665 PMC6037569

[5] Schultz C Engelhardt M. Anatomy of the hippocampal formation. Front Neurol Neurosci. (2014) 34:6–17. 10.1159/000360925 24777126

[6] Ghose S Chin R Gallegos A Roberts R Coyle J Tamminga C. Localization of NAAG-related gene expression deficits to the anterior hippocampus in schizophrenia. Schizophr Res. (2009) 111:131–7. 10.1016/j.schres.2009.03.038 19403271 PMC2685203

[7] Schobel SA Lewandowski NM Corcoran CM Moore H Brown T Malaspina D. Differential targeting of the CA1 subfield of the hippocampal formation by schizophrenia and related psychotic disorders. Arch Gen Psychiatry. (2009) 66:938–46. 10.1001/archgenpsychiatry.2009.115 19736350 PMC2797730

[8] Talati P Rane S Kose S Blackford JU Gore J Donahue MJ. Increased hippocampal CA1 cerebral blood volume in schizophrenia. Neuroimage Clin. (2014) 5:359–64. 10.1016/j.nicl.2014.07.004 25161901 PMC4141978

[9] Tamminga CA Stan AD Wagner AD. The hippocampal formation in schizophrenia. Am J Psychiatry. (2010) 167:1178–93. 10.1176/appi.ajp.2010.09081187 20810471

[10] Small SA Schobel SA Buxton RB Witter MP Barnes CA. A pathophysiological framework of hippocampal dysfunction in ageing and disease. Nat Rev Neurosci. (2011) 12:585–601. 10.1038/nrn3085 21897434 PMC3312472

[11] Clementz BA Sweeney JA Hamm JP Ivleva EI Ethridge LE Pearlson GD. Identification of distinct psychosis biotypes using brain-based biomarkers. Am J Psychiatry. (2016) 173:373–84. 10.1176/appi.ajp.2015.14091200 26651391 PMC5314432

[12] Haijma SV van Haren N Cahn W Koolschijn PC Hulshoff Pol HE Kahn RS. Brain volumes in schizophrenia: a meta-analysis in over 18 000 subjects. Schizophr Bull. (2013) 39:1129–38. 10.1093/schbul/sbs118 23042112 PMC3756785

[13] van Erp TG Hibar DP Rasmussen JM Glahn DC Pearlson GD Andreassen OA. Subcortical brain volume abnormalities in 2028 individuals with schizophrenia and 2540 healthy controls via the ENIGMA consortium. Mol Psychiatry. (2016) 21:547–53. 10.1038/mp.2015.63 26033243 PMC4668237

[14] Okada N Fukunaga M Yamashita F Koshiyama D Yamamori H Ohi K. Abnormal asymmetries in subcortical brain volume in schizophrenia. Mol Psychiatry. (2016) 21:1460–6. 10.1038/mp.2015.209 26782053 PMC5030462

[15] Yung AR Yuen HP McGorry PD Phillips LJ Kelly D Dell'Olio M. Mapping the onset of psychosis: the Comprehensive Assessment of At-Risk Mental States. Aust N Z J Psychiatry. (2005) 39:964–71. 10.1080/j.1440-1614.2005.01714.x 16343296

[16] Wood SJ Kennedy D Phillips LJ Seal ML Yücel M Nelson B. Hippocampal pathology in individuals at ultra-high risk for psychosis: a multi-modal magnetic resonance study. Neuroimage. (2010) 52:62–8. 10.1016/j.neuroimage.2010.04.012 20399273

[17] Dean DJ Orr JM Bernard JA Gupta T Pelletier-Baldelli A Carol EE. Hippocampal shape abnormalities predict symptom progression in neuroleptic-free youth at ultrahigh risk for psychosis. Schizophr Bull. (2016) 42:161–9. 10.1093/schbul/sbv086 26113620 PMC4681548

[18] Harrisberger F Buechler R Smieskova R Lenz C Walter A Egloff L. Alterations in the hippocampus and thalamus in individuals at high risk for psychosis. NPJ Schizophr. (2016) 2:16033. 10.1038/npjschz.2016.33 27738647 PMC5040554

[19] Velakoulis D Wood SJ Wong MT McGorry PD Yung A Phillips L. Hippocampal and amygdala volumes according to psychosis stage and diagnosis: a magnetic resonance imaging study of chronic schizophrenia, first-episode psychosis, and ultra-high-risk individuals. Arch Gen Psychiatry. (2006) 63:139–49. 10.1001/archpsyc.63.2.139 16461856

[20] Buehlmann E Berger GE Aston J Gschwandtner U Pflueger MO Borgwardt SJ. Hippocampus abnormalities in at risk mental states for psychosis? A cross-sectional high resolution region of interest magnetic resonance imaging study. J Psychiatr Res. (2010) 44:447–53. 10.1016/j.jpsychires.2009.10.008 19939408

[21] Witthaus H Mendes U Brüne M Ozgürdal S Bohner G Gudlowski Y. Hippocampal subdivision and amygdalar volumes in patients in an at-risk mental state for schizophrenia. J Psychiatry Neurosci. (2010) 35:33–40. 10.1503/jpn.090013 20040244 PMC2799502

[22] Klauser P Zhou J Lim JK Poh JS Zheng H Tng HY. Lack of evidence for regional brain volume or cortical thickness abnormalities in youths at clinical high risk for psychosis: findings from the longitudinal youth at risk study. Schizophr Bull. (2015) 41:1285–93. 10.1093/schbul/sbv012 25745033 PMC4601700

[23] Ho NF Holt DJ Cheung M Iglesias JE Goh A Wang M. Progressive decline in hippocampal CA1 volume in individuals at ultra-high-risk for psychosis who do not remit: findings from the longitudinal youth at risk study. Neuropsychopharmacology. (2017) 42:1361–70. 10.1038/npp.2017.5 28079061 PMC5437892

[24] Sasabayashi D Takayanagi Y Takahashi T Katagiri N Sakuma A Obara C. Subcortical brain volume abnormalities in individuals with an at-risk mental state. Schizophr Bull. (2020) 46:834–45. 10.1093/schbul/sbaa011 32162659 PMC7342178

[25] Ebdrup BH Skimminge A Rasmussen H Aggernaes B Oranje B Lublin H. Progressive striatal and hippocampal volume loss in initially antipsychotic-naive, first-episode schizophrenia patients treated with quetiapine: relationship to dose and symptoms. Int J Neuropsychopharmacol. (2011) 14:69–82. 10.1017/s1461145710000817 20701823

[26] Rizos E Papathanasiou MA Michalopoulou PG Laskos E Mazioti A Kastania A. A longitudinal study of alterations of hippocampal volumes and serum BDNF levels in association to atypical antipsychotics in a sample of first-episode patients with schizophrenia. PLoS ONE. (2014) 9:e87997. 10.1371/journal.pone.0087997 24551075 PMC3923760

[27] Lieberman J Chakos M Wu H Alvir J Hoffman E Robinson D. Longitudinal study of brain morphology in first episode schizophrenia. Biol Psychiatry. (2001) 49:487–99. 10.1016/s0006-3223(01)01067-8 11257234

[28] Wood SJ Velakoulis D Smith DJ Bond D Stuart GW McGorry PD. A longitudinal study of hippocampal volume in first episode psychosis and chronic schizophrenia. Schizophr Res. (2001) 52:37–46. 10.1016/s0920-9964(01)00175-x 11595390

[29] Whitworth AB Kemmler G Honeder M Kremser C Felber S Hausmann A. Longitudinal volumetric MRI study in first- and multiple-episode male schizophrenia patients. Psychiatry Res. (2005) 140:225–37. 10.1016/j.pscychresns.2005.07.006 16275040

[30] Mamah D Harms MP Barch D Styner M Lieberman JA Wang L. Hippocampal shape and volume changes with antipsychotics in early stage psychotic illness. Front Psychiatry. (2012) 3:96. 10.3389/fpsyt.2012.00096 23162479 PMC3495266

[31] Haukvik UK Tamnes CK Söderman E Agartz I. Neuroimaging hippocampal subfields in schizophrenia and bipolar disorder: a systematic review and meta-analysis. J Psychiatr Res. (2018) 104:217–26. 10.1016/j.jpsychires.2018.08.012 30107268

[32] Hu N Luo C Zhang W Yang X Xiao Y Sweeney JA. Hippocampal subfield alterations in schizophrenia: a selective review of structural MRI studies. Biomark Neuropsychiatry. (2020) 3:100026. 10.1016/j.bionps.2020.100026

[33] Ho NF Iglesias JE Sum MY Kuswanto CN Sitoh YY De Souza J. Progression from selective to general involvement of hippocampal subfields in schizophrenia. Mol Psychiatry. (2017) 22:142–52. 10.1038/mp.2016.4 26903271 PMC4995163

[34] Mathew I Gardin TM Tandon N Eack S Francis AN Seidman LJ. Medial temporal lobe structures and hippocampal subfields in psychotic disorders: findings from the Bipolar-Schizophrenia Network on Intermediate Phenotypes (B-SNIP) study. JAMA Psychiatry. (2014) 71:769–77. 10.1001/jamapsychiatry.2014.453 24828364

[35] Haukvik UK Westlye LT Mørch-Johnsen L Jørgensen KN Lange EH Dale AM. *In vivo* hippocampal subfield volumes in schizophrenia and bipolar disorder. Biol Psychiatry. (2015) 77:581–8. 10.1016/j.biopsych.2014.06.020 25127742

[36] Vargas T Dean DJ Osborne KJ Gupta T Ristanovic I Ozturk S. Hippocampal subregions across the psychosis spectrum. Schizophr Bull. (2018) 44:1091–9. 10.1093/schbul/sbx160 29272467 PMC6101630

[37] Wisse LE Biessels GJ Geerlings MI. A critical appraisal of the hippocampal subfield segmentation package in FreeSurfer. Front Aging Neurosci. (2014) 6:261. 10.3389/fnagi.2014.00261 25309437 PMC4174865

[38] Kuhn S Musso F Mobascher A Warbrick T Winterer G Gallinat J. Hippocampal subfields predict positive symptoms in schizophrenia: first evidence from brain morphometry. Transl Psychiatry. (2012) 2:e127. 10.1038/tp.2012.51 22692142 PMC3384220

[39] Kawano M Sawada K Shimodera S Ogawa Y Kariya S Lang DJ. Hippocampal subfield volumes in first episode and chronic schizophrenia. PLoS ONE. (2015) 10:e0117785. 10.1371/journal.pone.0117785 25658118 PMC4319836

[40] Nakahara S Turner JA Calhoun VD Lim KO Mueller B Bustillo JR. Dentate gyrus volume deficit in schizophrenia. Psychol Med. (2020) 50:1267–77. 10.1017/s0033291719001144 31155012 PMC7068799

[41] Iglesias JE Augustinack JC Nguyen K Player CM Player A Wright M. A computational atlas of the hippocampal formation using *ex vivo*, ultra-high resolution MRI: application to adaptive segmentation of *in vivo* MRI. Neuroimage. (2015) 115:117–37. 10.1016/j.neuroimage.2015.04.042 25936807 PMC4461537

[42] Leemput KV Bakkour A Benner T Wiggins G Wald LL Augustinack J. Automated segmentation of hippocampal subfields from ultra-high resolution *in vivo* MRI. Hippocampus. (2009) 19:549–57. 10.1002/hipo.20615 19405131 PMC2739884

[43] Sone D Sato N Maikusa N Ota M Sumida K Yokoyama K. Automated subfield volumetric analysis of hippocampus in temporal lobe epilepsy using high-resolution T2-weighed MR imaging. Neuroimage Clin. (2016) 12:57–64. 10.1016/j.nicl.2016.06.008 27489767 PMC4960104

[44] Hou CL Xiang YT Wang ZL Everall I Tang Y Yang C. Cognitive functioning in individuals at ultra-high risk for psychosis, first-degree relatives of patients with psychosis and patients with first-episode schizophrenia. Schizophr Res. (2016) 174:71–6. 10.1016/j.schres.2016.04.034 27197904

[45] First MB Gibbon M Spitzer RL Williams JBW. Structured Clinical Interview for DSM-IV Axis I Disorders. Washington DC: American Psychiatric Press. (1997).

[46] American Psychiatric Association. Diagnostic and Statistical Manual of Mental Disorders. 4th ed. Text Revision. Washington DC: American Psychiatric Association Press (2000).

[47] American Psychiatric Association. Diagnostic and Statistical Manual of Mental Disorders. 5th ed. Washington DC: American Psychiatric Association Press (2013).

[48] Flaum MA Andreasen NC Arndt S. The Iowa prospective longitudinal study of recent-onset psychoses. Schizophr Bull. (1992) 18:481–90. 10.1093/schbul/18.3.481 1411335

[49] Breitborde NJK Srihari VH Wood SW. Review of the operational definition for first-episode psychosis. Early Interv Psychiatry. (2009) 3:259–65. 10.1001/archpsyc.57.7.692 22642728 PMC4451818

[50] Takahashi T Kido M Sasabayashi D Nakamura M Furuichi A Takayanagi Y. Gray matter changes in the insular cortex during the course of the schizophrenia spectrum. Front Psychiatry. (2020) 11:659. 10.3389/fpsyt.2020.00659 32754066 PMC7366364

[51] Mizuno M Suzuki M Matsumoto K Murakami M Takeshi K Miyakoshi T. Clinical practice and research activities for early psychiatric intervention at Japanese leading centres. Early Interv Psychiatry. (2009) 3:5–9. 10.1111/j.1751-7893.2008.00104.x 21352169

[52] Miyakoshi T Matsumoto K Ito F Ohmuro N Matsuoka H. Application of the Comprehensive Assessment of At-Risk Mental States (CAARMS) to the Japanese population: reliability and validity of the Japanese version of the CAARMS. Early Interv Psychiatry. (2009) 3:123–30. 10.1111/j.1751-7893.2009.00118.x 21352185

[53] International Early Psychosis Association Writing Group. International clinical practice guidelines for early psychosis. Br J Psychiatry Suppl. (2005) 48:s120–4. 10.1192/bjp.187.48.s120 16055801

[54] Takahashi T Tsugawa S Nakajima S Plitman E Chakravarty MM Masuda F. Thalamic and striato-pallidal volumes in schizophrenia patients and individuals at risk for psychosis: a multi-atlas segmentation study. Schizophr Res. (2020). 10.1016/j.schres.2020.04.016. [Epub ahead of print]. 32448678

[55] Kay SR Fiszbein A Opler LA. The positive and negative syndrome scale (PANSS) for schizophrenia. Schizophr Bull. (1987) 13:261–76. 10.1093/schbul/13.2.261 3616518

[56] Keefe RS Goldberg TE Harvey PD Gold JM Poe MP Coughenour L. The Brief Assessment of Cognition in Schizophrenia: reliability, sensitivity, and comparison with a standard neurocognitive battery. Schizophr Res. (2004) 68:283–97. 10.1016/j.schres.2003.09.011 15099610

[57] Kaneda Y Sumiyoshi T Keefe R Ishimoto Y Numata S Ohmori T. Brief assessment of cognition in schizophrenia: validation of the Japanese version. Psychiatry Clin Neurosci. (2007) 61:602–9. 10.1111/j.1440-1819.2007.01725.x 18081619

[58] Kaneda Y Omori T Okahisa Y Sumiyoshi T Pu S Ueoka Y. Measurement and treatment research to improve cognition in schizophrenia consensus cognitive battery: validation of the Japanese version. Psychiatry Clin Neurosci. (2013) 67:182–8. 10.1111/pcn.12029 23581870

[59] Keefe RS Poe M Walker TM Kang JW Harvey PD. The Schizophrenia Cognition Rating Scale: an interview-based assessment and its relationship to cognition, real-world functioning, and functional capacity. Am J Psychiatry. (2006) 163:426–32. 10.1176/appi.ajp.163.3.426 16513863

[60] Kaneda Y Ueoka Y Sumiyoshi T Yasui-Furukori N Ito T Higuchi Y. Schizophrenia Cognition Rating Scale Japanese version (SCoRS-J) as a co-primary measure assessing cognitive function in schizophrenia. Nihon Shinkei Seishin Yakurigaku Zasshi. (2011) 31:259–62. 22256616

[61] Higuchi Y Sumiyoshi T Seo T Suga M Takahashi T Nishiyama S. Associations between daily living skills, cognition, and real-world functioning across stages of schizophrenia; a study with the Schizophrenia Cognition Rating Scale Japanese version. Schizophr Res Cogn. (2017) 7:13–18. 10.1016/j.scog.2017.01.001 28740824 PMC5514300

[62] Goldman HH Skodol AE Lave TR. Revising axis V for DSM-IV: a review of measures of social functioning. Am J Psychiatry. (1992) 149:1148–56. 10.1176/ajp.149.9.1148 1386964

[63] Sled JG Zijdenbos AP Evans AC. A nonparametric method for automatic correction of intensity nonuniformity in MRI data. IEEE Trans Med Imaging. (1998) 17:87–97. 10.1109/42.668698 9617910

[64] Dale AM Fischl B Sereno MI. Cortical surface-based analysis. I. Segmentation and surface reconstruction. Neuroimage. (1999) 9:179–94. 10.1006/nimg.1998.0395 9931268

[65] Fischl B Sereno MI Dale AM. Cortical surface-based analysis. II: Inflation, flattening, and a surface-based coordinate system. Neuroimage. (1999) 9:195–207. 10.1006/nimg.1998.0396 9931269

[66] Brown EM Pierce ME Clark DC Fischl BR Iglesias JE Milberg WP. Test-retest reliability of FreeSurfer automated hippocampal subfield segmentation within and across scanners. Neuroimage. (2020) 210:116563. 10.1016/j.neuroimage.2020.116563 31972281

[67] Chiappiniello A Tarducci R Muscio C Bruzzone MG Bozzali M Tiraboschi P. Automatic multispectral MRI segmentation of human hippocampal subfields: an evaluation of multicentric test-retest reproducibility. Brain Struct Funct. (2021) 226:137–50. 10.1007/s00429-020-02172-w 33231744 PMC7817563

[68] Joie RL Fouquet M Mezenge F Landeau B Villain N Mevel K. Differential effect of age on hippocampal subfields assessed using a new high-resolution 3T MR sequence. NeuroImage. (2010) 53:506–14. 10.1016/j.neuroimage.2010.06.024 20600996

[69] Flores R Joie RL Landeau B Perrotin A Mezenge F Sayette V. Effects of age and Alzheimer's disease on hippocampal subfields: comparison between manual and FreeSurfer volumetry. Hum Brain Mapp. (2015) 36:463–74. 10.1002/hbm.22640 25231681 PMC6869780

[70] Baglivo V Cao B Mwangi B Bellani M Perlini C Lasalvia A. Hippocampal subfield volumes in patients with first-episode psychosis. Schizophr Bull. (2018) 44:552–9. 10.1093/schbul/sbx108 29897598 PMC5890476

[71] Simić G Kostović I Winblad B Bogdanović N. Volume and number of neurons of the human hippocampal formation in normal aging and Alzheimer's disease. J Comp Neurol. (1997) 379:482–94. 10.1002/(sici)1096-9861(19970324)379:4<482::aid-cne2>3.0.co;2-z 9067838

[72] Lim HK Hong SC Jung WS Ahn KJ Won WY Hahn C. Automated segmentation of hippocampal subfields in drug-naïve patients with Alzheimer disease. AJNR Am J Neuroradiol. (2013) 34:747–51. 10.3174/ajnr.A3293 23042923 PMC7964501

[73] Balu DT Lucki I. Adult hippocampal neurogenesis: regulation, functional implications, and contribution to disease pathology. Neurosci Biobehav Rev. (2009) 33:232–52. 10.1016/j.neubiorev.2008.08.007 18786562 PMC2671071

[74] Schmitt A Weber S Jatzko A Braus DF Henn FA. Hippocampal volume and cell proliferation after acute and chronic clozapine or haloperidol treatment. J Neural Transm. (2004) 111:91–100. 10.1007/s00702-003-0070-2 14714218

[75] Hu N Sun H Fu G Zhang W Xiao Y Zhang L. Anatomic abnormalities of hippocampal subfields in never-treated and antipsychotic-treated patients with long-term schizophrenia. Neuropsychopharmacol. (2020) 35:39–48. 10.1016/j.euroneuro.2020.03.020 32402652

[76] Schobel SA Chaudhury NH Khan UA Paniagua B Styner MA Asllani I. Imaging patients with psychosis and a mouse model establishes a spreading pattern of hippocampal dysfunction and implicates glutamate as a driver. Neuron. (2013) 78:81–93. 10.1016/j.neuron.2013.02.011 23583108 PMC3966570

[77] Gozzi A Herdon H Schwarz A Bertani S Crestan V Turrini G. Pharmacological stimulation of NMDA receptors via co-agonist site suppresses fMRI response to phencyclidine in the rat. Psychopharmacology. (2008) 201:273–84. 10.1007/s00213-008-1271-z 18704372

[78] Javitt DC Freedman R. Sensory processing dysfunction in the personal experience and neuronal machinery of schizophrenia. Am J Psychiatry. (2015) 172:17–31. 10.1176/appi.ajp.2014.13121691 25553496 PMC4501403

[79] Javitt DC. Glycine transport inhibitors for the treatment of schizophrenia: symptom and disease modification. Curr Opin Drug Discov Devel. (2009) 12:468–78. 19562643

[80] Javitt DC. Biotypes in psychosis: has the RDoC era arrived? Am J Psychiatry. (2016) 173:313–4. 10.1176/appi.ajp.2016.16020140 27035527

[81] Eack SM Hogarty GE Cho RY Prasad KM Greenwald DP Hogarty SS. Neuroprotective effects of cognitive enhancement therapy against gray matter loss in early schizophrenia: results from a 2-year randomized controlled trial. Arch Gen Psychiatry. (2010) 67:674–82. 10.1001/archgenpsychiatry.2010.63 20439824 PMC3741671

[82] Lin J Chan SK Lee EH Chang WC Tse M Su WW. Aerobic exercise and yoga improve neurocognitive function in women with early psychosis. NPJ Schizophr. (2015) 1:15047. 10.1038/npjschz.2015.47 27336050 PMC4849465

[83] Heckers S Konradi C. GABAergic mechanisms of hippocampal hyperactivity in schizophrenia. Schizophr Res. (2015) 167:4–11. 10.1016/j.schres.2014.09.041 25449711 PMC4402105

[84] Lisman JE Coyle JT Green RW Javitt DC Benes FM Heckers S. Circuit-based framework for understanding neurotransmitter and risk gene interactions in schizophrenia. Trends Neurosci. (2008) 31:234–42. 10.1016/j.tins.2008.02.005 18395805 PMC2680493

[85] Coultrap SJ Nixon KM Alvestad RM Valenzuela CF Browning MD. Differential expression of NMDA receptor subunits and splice variants among the CA1, CA3 and dentate gyrus of the adult rat. Brain Res Mol Brain Res. (2005) 135:104–11. 10.1016/j.molbrainres.2004.12.005 15857673

[86] Newell DW Malouf AT Franck JE. Glutamate-mediated selective vulnerability to ischemia is present in organotypic cultures of hippocampus. Neurosci Lett. (1990) 116:325–30. 10.1016/0304-3940(90)90095-q 1978744

[87] Kraguljac NV White DM Reid MA Lahti AC. Increased hippocampal glutamate and volumetric deficits in unmedicated patients with schizophrenia. JAMA Psychiatry. (2013) 70:1294–302. 10.1001/jamapsychiatry.2013.2437 24108440 PMC7891898

[88] Benes FM. Evidence for altered trisynaptic circuitry in schizophrenic hippocampus. Biol Psychiatry. (1999) 46:589–99. 10.1016/s0006-3223(99)00136-5 10472413

[89] Benes FM Berretta S. GABAergic interneurons: implications for understanding schizophrenia and bipolar disorder. Neuropsychopharmacology. (2001) 25:1–27. 10.1016/s0893-133x(01)00225-1 11377916

[90] Friston KJ Liddle PF Frith CD Hirsch SR Frackowiak RS. The left medial temporal region and schizophrenia. A PET study. Brain. (1992) 115:367–82. 10.1093/brain/115.2.367 1606474

[91] Dierks T Linden DE Jandl M Formisano E Goebel R Lanfermann H. Activation of Heschl's gyrus during auditory hallucinations. Neuron. (1999) 22:615–21. 10.1016/s0896-6273(00)80715-1 10197540

[92] Schwarcz R Witter MP. Memory impairment in temporal lobe epilepsy: the role of entorhinal lesions. Epilepsy Res. (2002) 50:161–77. 10.1016/s0920-1211(02)00077-3 12151126

[93] Grimm CM Aksamaz S Schulz S Teutsch J Sicinski P Liss B. Schizophrenia-related cognitive dysfunction in the Cyclin-D2 knockout mouse model of ventral hippocampal hyperactivity. Transl Psychiatry. (2018) 8:212. 10.1038/s41398-018-0268-6 30301879 PMC6178344

[94] Toro C Deakin JFW. NMDA receptor subunit NRI and postsynaptic protein PSD-95 in hippocampus and orbitofrontal cortex in schizophrenia and mood disorder. Schizophr Res. (2005) 80:323–30. 10.1016/j.schres.2005.07.003 16140506

[95] Hýža M Huttlová J Kerkovský M Kašpárek T. Psychosis effect on hippocampal reduction in schizophrenia. Prog Neuropsychopharmacol Biol Psychiatry. (2014) 48:186–92. 10.1016/j.pnpbp.2013.10.008 24140928

[96] Lodge DJ Grace AA. Gestational methylazoxymethanol acetate administration: a developmental disruption model of schizophrenia. Behav Brain Res. (2009) 204:306–12. 10.1016/j.bbr.2009.01.031 19716984 PMC2736136

[97] Sanderson TM Cotel MC O'Neill MJ Tricklebank MD Collingridge GL Sher E. Alterations in hippocampal excitability, synaptic transmission and synaptic plasticity in a neurodevelopmental model of schizophrenia. Neuropharmacology. (2012) 62:1349–58. 10.1016/j.neuropharm.2011.08.005 21854789

[98] Winterburn JL Pruessner JC Chavez S Schira MM Lobaugh NJ Voineskos AN. A novel *in vivo* atlas of human hippocampal subfields using high-resolution 3 T magnetic resonance imaging. Neuroimage. (2013) 74:254–65. 10.1016/j.neuroimage.2013.02.003 23415948

[99] Yang C Wu S Lu W Bai Y Gao H. Brain differences in first-episode schizophrenia treated with quetiapine: a deformation-based morphometric study. Psychopharmacology. (2015) 232:369–77. 10.1007/s00213-014-3670-7 25080851

[100] Cha J Greenberg T Song I Simpson HB Posner J Mujica-Parodi LR. Abnormal hippocampal structure and function in clinical anxiety and comorbid depression. Hippocampus. (2016) 26:545–53. 10.1002/hipo.22566 26743454 PMC4837065

[101] Sala M Perez J Soloff P di Nemi SU Caverzasi E Soares JC. Stress and hippocampal abnormalities in psychiatric disorders. Eur Neuropsychopharmacol. (2004) 14:393–405. 10.1016/j.euroneuro.2003.12.005 15336301

[102] Chen LW Sun D Davis SL Haswell CC Dennis EL Swanson CA. Smaller hippocampal CA1 subfield volume in posttraumatic stress disorder. Depress Anxiety. (2018) 35:1018–29. 10.1002/da.22833 30256497 PMC6261348

[103] Han KM Kim A Kang W Kang Y Kang J Won E. Hippocampal subfield volumes in major depressive disorder and bipolar disorder. Eur Psychiatry. (2019) 57:70–7. 10.1016/j.eurpsy.2019.01.016 30721801

