# Supplementary material for: Reduced Hippocampal Subfield Volume in Schizophrenia and Clinical High-Risk State for Psychosis
Source: Front Psychiatry. 2021 Mar 22;12:642048. doi: 10.3389/fpsyt.2021.642048 (PMC8019805; doi:10.3389/fpsyt.2021.642048)
Supplement: Supplementary file 1 [file Data_Sheet_1.docx]

**Supplementary Material**

**Supplementary Table S1.** Absolute volume of the hippocampal subfields in the HC group and ARMS subgroup without comorbid PDD diagnosis

**Supplementary Table S2.** Absolute volume of the hippocampal subfields in the ARMS-NP and ARMS-P groups

**Supplementary Table S3.** Absolute volume of the hippocampal subfields in the ROSz and chronic Sz groups

**Supplementary Table S4.** Absolute volume of the hippocampal subfields in the ROSz and re-defined chronic Sz groups

**Supplementary Table S5.** Absolute volume of the hippocampal subfields in the ARMS, ROSz, and chronic Sz groups

**Supplementary Table S6.** Absolute volume of the merged hippocampal subfields in the HC, ARMS, and Sz groups
